# Supplementary material for: Observation of Enhanced Dissociative Photochemistry in the Non-Native Nucleobase 2-Thiouracil
Source: Molecules. 2020 Jul 10;25(14):3157. doi: 10.3390/molecules25143157 (PMC7397253; doi:10.3390/molecules25143157)
Supplement: Supplementary file 1 [file molecules-25-03157-s001.pdf]

Supplementary Material

Observation of Enhanced Dissociative Photochemistry in the Non-native Nucleobase 2-Thiouracil

Kelechi O. Uleanya, Rosaria Cercola, Maria Nikolova, Edward Matthews, Natalie G.K. Wong, and Caroline E.H. Dessent*

Department of Chemistry, University of York, Heslington, York, YO10 5DD, UK.

Contents

S1. Time dependent density functional theory data of all tautomers of deprotonated and protonated 2-thiouracil

S2. Electron detachment spectrum of deprotonated thiouracil

S3. Higher collisional dissociation of deprotonated and protonated 2-thiouracil

S4. Optimized fragments production energies relative energies to parent ion energy

S5. Photofragment difference (laseron-laseroff) mass spectra of protonated 2-thiouracil excited at (a) 4.6 eV (269nm) and 5.2 eV (238nm)

S6. Molecular orbital transitions in deprotonated and protonated 2-thiouracil

Section S1: Time dependent density functional theory data of all tautomers of deprotonated and protonated 2-thiouracil

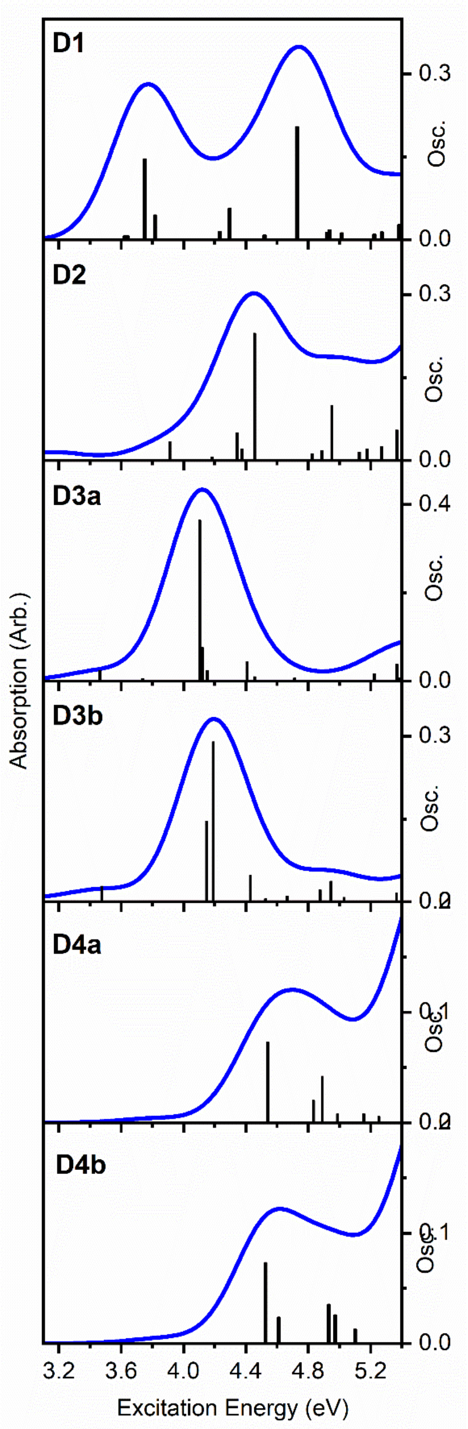

Fig. S1 Calculated TDDFT excitation energies (with the B3LYP/6- 311++G (2d, 2p) functional and basis set) of D1,D2,D3a,D3b,D4a and D4b tautomer of [2-TU - H]$^-$ The oscillator strengths (OSC.) on the y axis of individual transitions ≥ 0.005 within the experimental scan range are shown by vertical bars, while the full line spectrum is a convolution of the calculated spectrum with Gaussian function (0.25 eV HWHM)

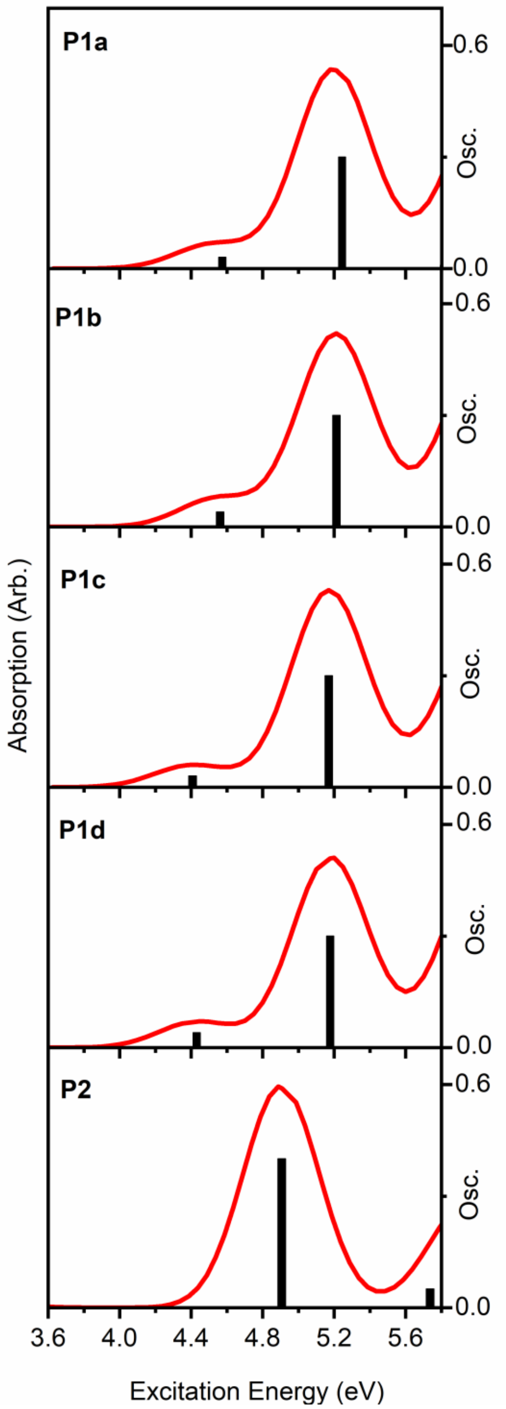

Fig. S2 Calculated TDDFT excitation energies (with the B3LYP/6- 311++G (2d, 2p) functional and basis set) of P1a, P1b, P1c, P1d and P2 tautomer of [2-TU · H] $^{+}$. The oscillator strengths (OSC.) on the y axis of individual transitions within the experimental scan range are shown by vertical bars, while the full line spectrum is convolution the calculated spectrum with Gaussian function (0.25eV HWHM).

Section S2: Electron detachment spectrum of deprotonated thiouracil

Electron detachment (ED) yield of [2-TU - H]$^-$ ion is displayed in Fig S3. Although electron loss cannot be directly measured in our instrument, we calculate it by assuming that any photodepleted ions that is not detected as an ionic-fragments are electron loss.

Note that our instrument can only detect ions with m/z >50

ED = (Photodepletion ion count -∑Photofragment ion count)

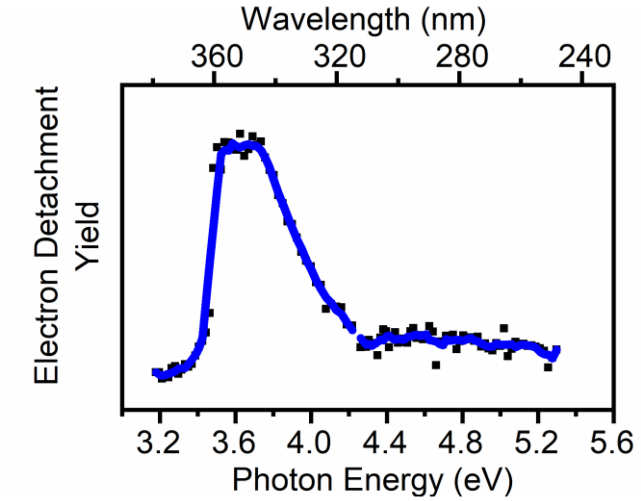

Fig. S3 Electron detachment yield of mass selected [2-TU - H]$^-$ ions, across the range 3.2-5.2 eV (234-390nm). The solid line is a five-point adjacent average of data points

Section S3: Higher energy collisional dissociation of deprotonated and protonated 2-thiouracil

Higher energy collisional dissociation (HCD) was performed on isolated deprotonated and protonated 2-thiouracil to determine the ground state thermal fragments. Fig. S4 and S5 displays as a function of applied % HCD energy, the relative intensities of the deprotonated and protonated 2-thiouracil parent ion and fragments production intensities respectively.

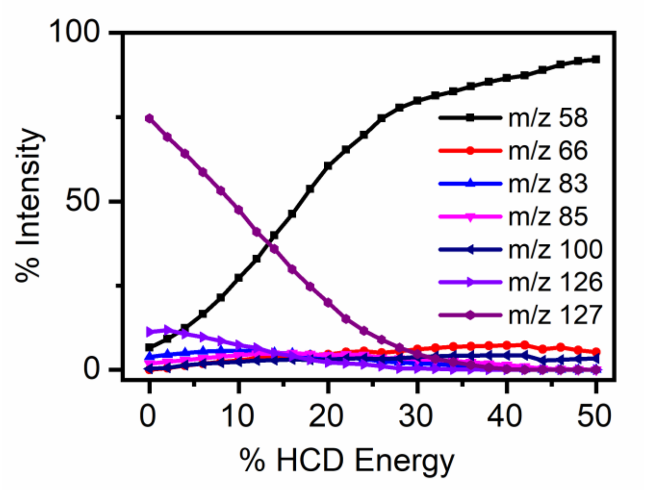

Fig. S4 Parent ion dissociation curve [2-TU - H]$^-$ alongside production curves of 6 most intense fragments upon HCD between 0 and 50% energy. The data points fitted with the curved lines are viewing guides to show the profile for an individual fragment.

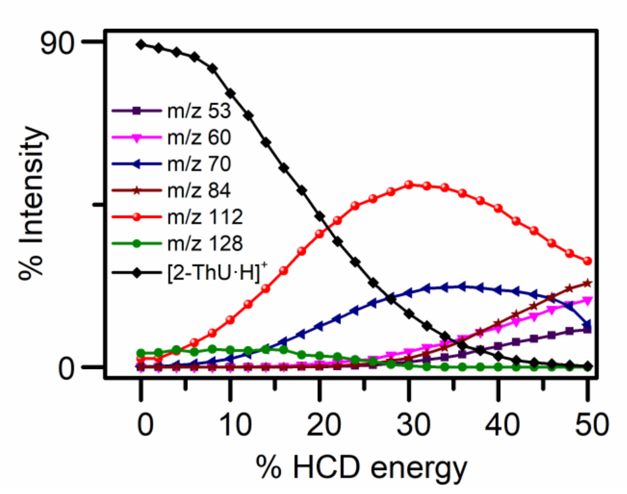

Fig. S5 Parent ion dissociation curve [2-TU · H]$^+$ alongside production curves of five most intense fragments upon HCD between 0 and 50% energy. The data points fitted with the curved lines are viewing guides to show the profile for an individual fragment.

Section S4: Relative energies of fragments for each fragmentation pathway to parent ion energy

The relative energies are calculated with a comparison of the sum of the zero point energies of the ionic fragments and the neutral or radical fragments with the parent ion zero-point energy. In the secondary fragmentation pathway, the comparison is done with the energy of the ionic parent fragment. This is done with an assumption that the global minima energies of the fragments could give insight into their stability.

Parent ion (E) - (Fragment A(E) + Fragment B (E)) = Relative energies

Where E = zero-point energy, fragment A = ionic fragment and fragment B = neutral or radical fragment.

Table S1: Relative energies calculated at B3LYP/6- 311++G (2d, 2p) (gas phase) for the major fragmentation pathways of $[2\text{-TU - H}]^-$. All energies are zero point corrected.

	Rel. Energies (eV)
D1 tautomer	0.0
Thiocyanate + Acrylamide	-0.48
Thiocyanate + 3-aminopropan-2-enal	0.207

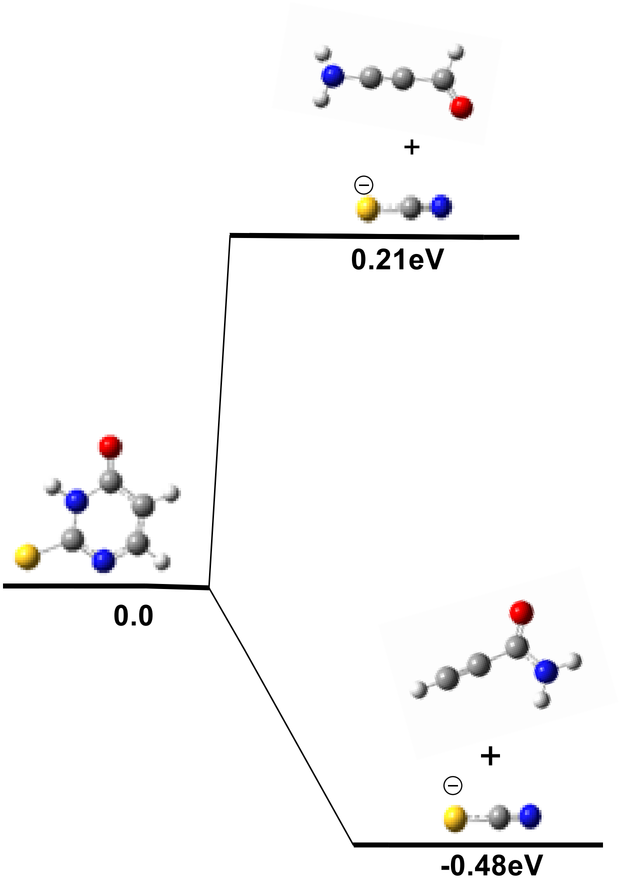

Fig. S6 Energy diagram for relative energies calculated at B3LYP/6- 311++G (2d, 2p) (gas phase) for the fragmentation pathways of [2-TU - H]$^-$

Table S2: Step 1 relative energies calculated at B3LYP/6- 311++G (2d, 2p) (gas phase) for the major fragmentation pathways of [2-TU · H]$^+$. All energies are zero point corrected.

	Rel. Energies (eV)
P1 tautomer	0.0
m/z 112 + NH3	0.21
m/z 70 + HNCS	0.1

Table S3: Step 2 relative energies calculated at B3LYP/6- 311++G (2d, 2p) (gas phase) for the possible fragments in the secondary fragmentation pathways of [2-TU · H]$^+$ through the ionic fragments m/z 112(A) and m/z 70(B).

A

	Rel. Energies (eV)
m/z 112	0.0
CO + m/z 84	0.1
m/z 52 +m/z 60	0.5
m/z 59 + m/z 53	0.17

B

	Rel. Energies (eV)
m/z 70	0.0
m/z 53 +NH_3	0.29

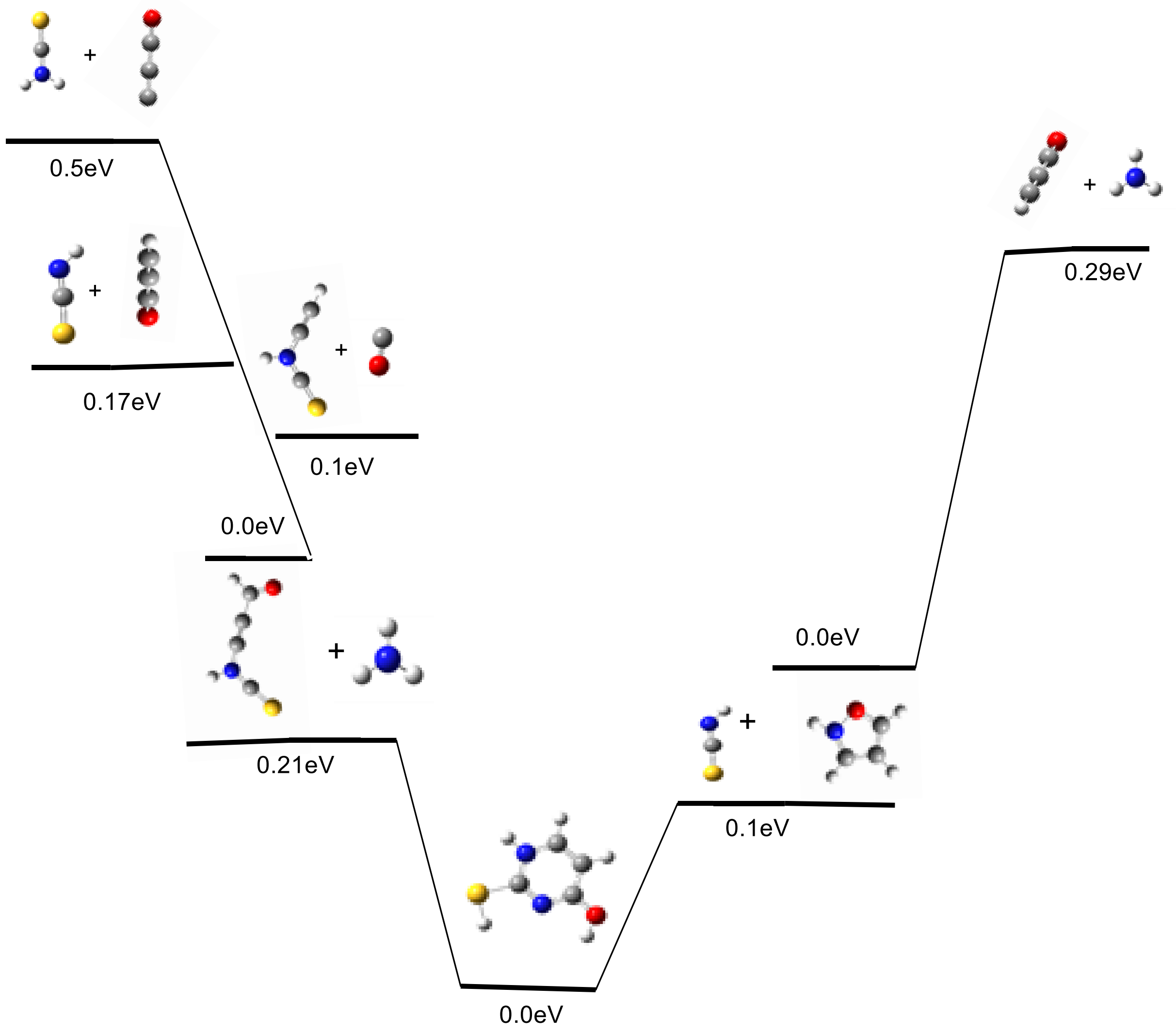

Fig. S7 Energy diagram for relative energies calculated at B3LYP/6- 311++G (2d, 2p) (gas phase) for the major and the secondary fragmentation pathways of [2-TU · H]$^+$

Section S5: Photofragment difference ($laser_{on}$-$laser_{off}$) mass spectra of $[2\text{-TU} \cdot H]^+$, excited at (a) 4.6 eV (269nm) and 5.2 eV (238nm).

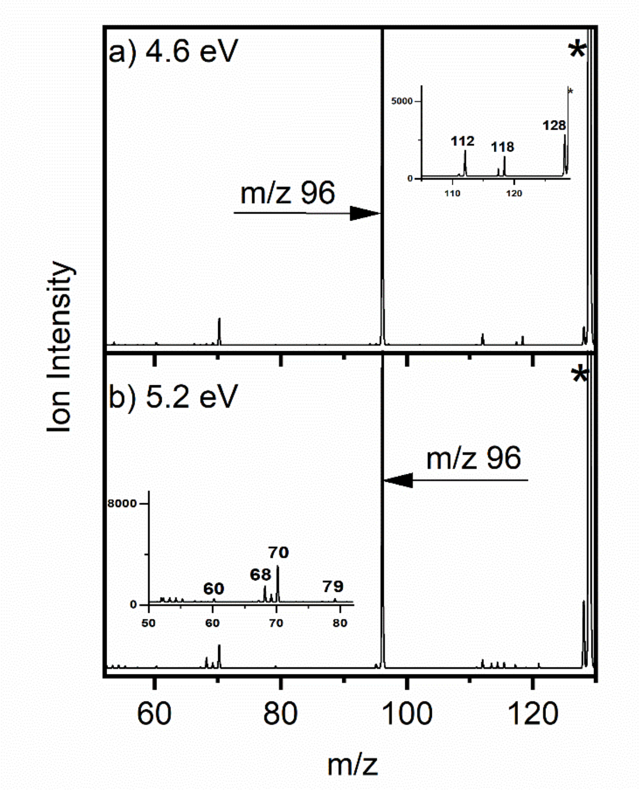

Fig. S8 Photofragment difference ($laser_{on}$-$laser_{off}$) mass spectra of $[2\text{-TU}{\cdot}H]^+$, excited at (a) 4.6 eV (269nm) and 5.2 eV (238nm). *Represents parent ion signal.

Section S6: Molecular orbital transitions in deprotonated and protonated 2-thiouracil

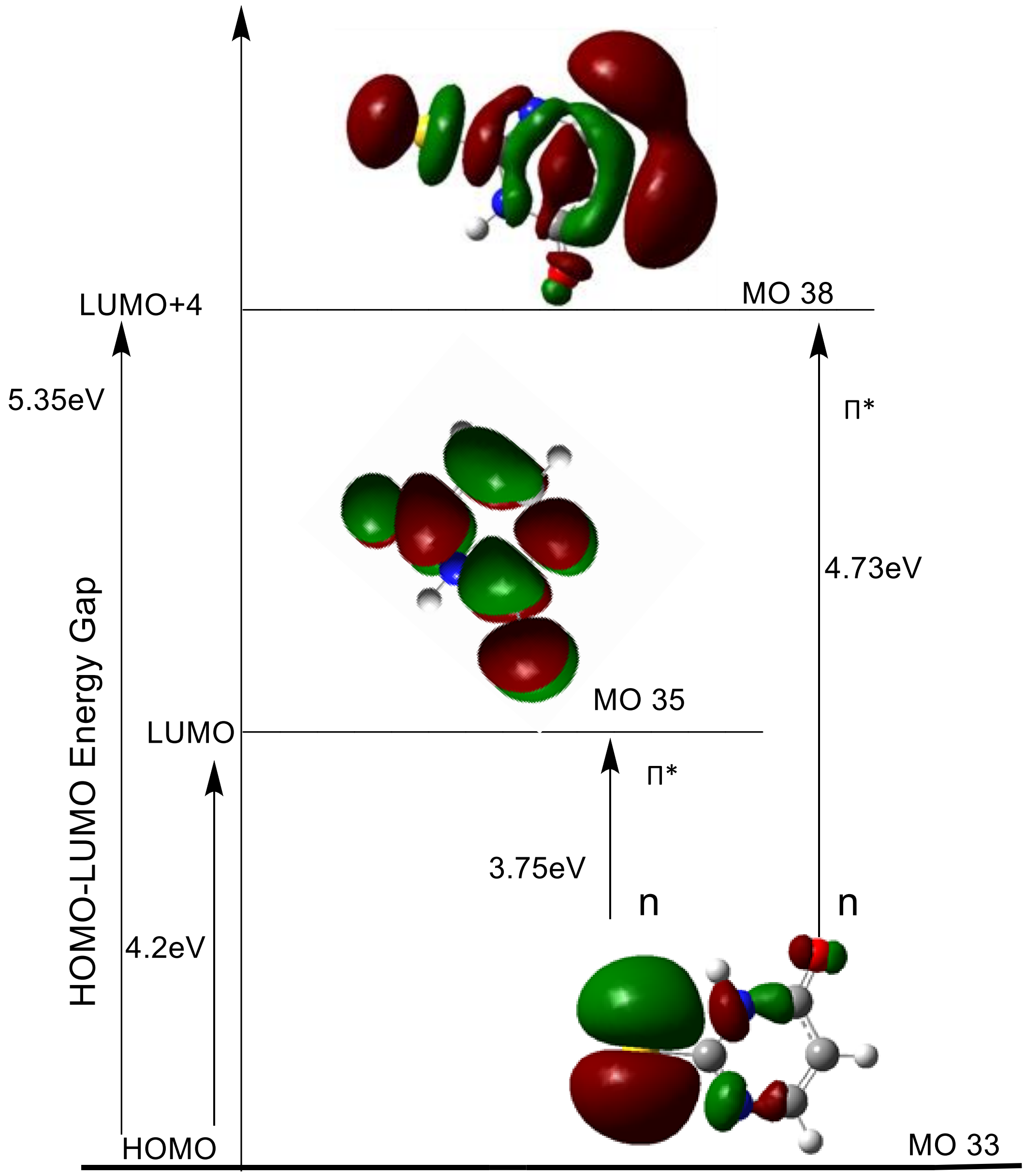

Fig. S9: Molecular orbital transitions predicted by TDDFT calculations at 3.75 and 4.73eV for D1 of $[2\text{-TU - H}]^-$

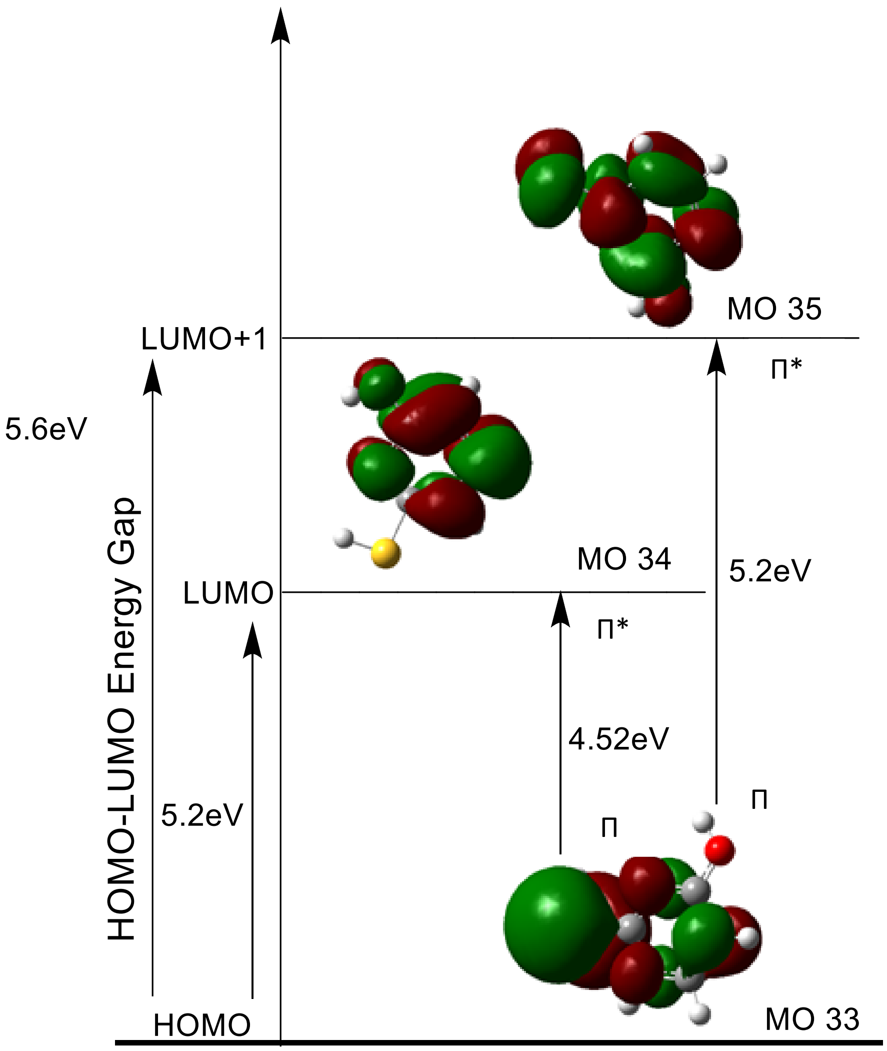

Fig. S10: Molecular orbital transitions predicted by TDDFT calculations at 4.52 and 5.2eV for P1a of [2-TU · H]$^+$

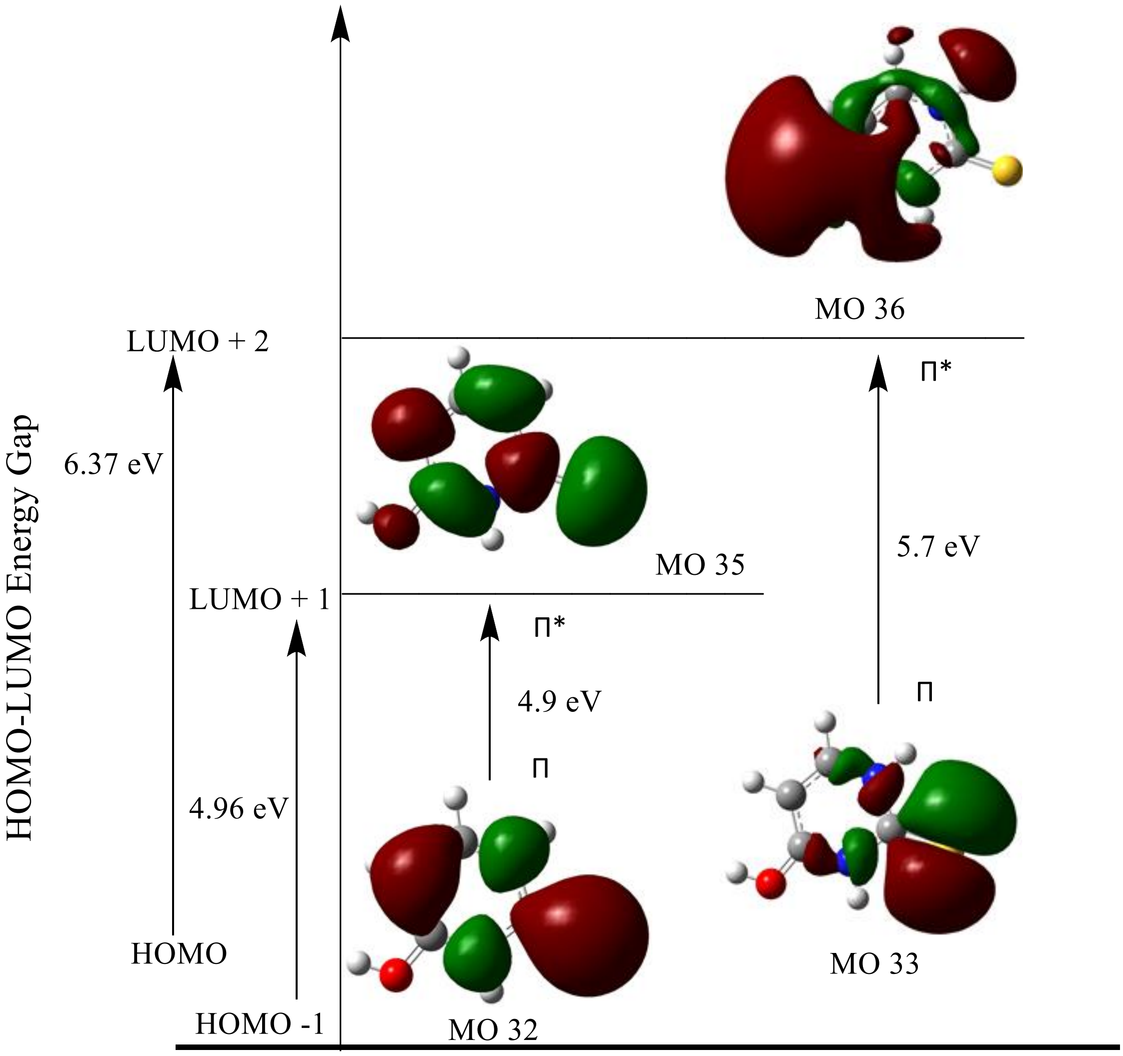

Fig. S11. Molecular orbital transitions predicted by TDDFT calculations at 4.9 and 5.7 eV for [2-TU · H]$^+$ P2 optimised geometric structure.
